# Return to Sport and Risk of Second Anterior Cruciate Ligament (ACL) Injury in Elite Male Footballers: 10-Year Experience From a Single Center

**DOI:** 10.1177/23259671251391362

**Published:** 2025-12-03

**Authors:** Alberto Grassi, Tommaso Bonanzinga, Federico Maria Adravanti, Gian Andrea Lucidi, Emanuele Altovino, Stefano Zaffagnini, Maurilio Marcacci

**Affiliations:** * II Clinica Ortopedica e Traumatologica, IRCCS Istituto Ortopedico Rizzoli, Bologna, Italy; † Dipartimento di Scienze Biomediche e Neuromotorie (DIBINEM), University of Bologna, Bologna, Italy; ‡ Department of Biomedical Sciences, Humanitas University, Milan, Italy; § IRCCS Humanitas Research Hospital, Rozzano, Milan, Italy; Investigation performed at Clinica Ortopedica e Traumatologica II, IRCCS (Istituto di Ricovero e Cura a Carattere Scientifico) Istituto Ortopedico Rizzoli, Bologna, Italy

**Keywords:** anterior cruciate ligament, knee, professional athletes, return to play, soccer, sports trauma

## Abstract

**Background:**

Anterior cruciate ligament (ACL) injury among professional footballers represents a career-threatening injury with a high burden for athletes and teams. Debated issues in professional footballers after ACL reconstruction (ACLR) include time to return to play (RTP), level of play after surgery, and incidence and timing of sustaining a second ACL injury.

**Purposes:**

To analyze RTP and performance 2 seasons after surgery of professional footballers who underwent ACLR, to evaluate factors influencing the timing of RTP and second injury risk, and to assess the level of RTP and performance after surgery.

**Study Design:**

Case series; Level of evidence, 4.

**Methods:**

All professional male footballers who underwent ACLR using a hamstrings graft with or without a lateral extra-articular procedure at a single institution between 2002 and 2013 were retrospectively included. Demographic and intraoperative findings were extracted from the medical records. Data regarding the number of matches played in 5 seasons (injury season, the 2 seasons before, and the 2 seasons after the injury), the level of play in those 5 seasons, the time to return to the first official match, and reinjuries were extracted from publicly available databases, and patients were contacted to confirm. Univariate and multivariate analyses were conducted to identify factors influencing RTP and risk factors for second ACL injury.

**Results:**

A total of 58 primary ACLRs were included; 20 (34.5%) participated in the first division (Serie A). RTP was 96.6% (56/58) after a mean of 6.4 ± 2.3 months, and return to match occurred after a mean of 7.5 ± 3.3 months. Patients without meniscal injury had a faster RTP (5.6 ± 1.6 months; *P* = .0016), while athletes with cartilage injury showed a longer time to RTP (10.2 ± 3.7 months; *P* = .0001) and resulted as a predictor for longer RTP (+4.4 months; *P* = .0047).

In the second season after an ACL injury, 23% of footballers participated in a lower division with respect to the injury season, playing a mean of 20 ± 11 matches, with 65% of them participating in >20 games. The second injury rate was 15.8% (9 patients). The ipsilateral reoperation rate, including ACL revisions, was 28.1%. Additionally, returning to competitive play within 5 months of surgery was a significant predictor of a second ACL injury.

**Conclusion:**

Almost all patients returned to play after ACLR using hamstrings graft with over-the-top techniques, typically regaining preinjury performance by the second season after the injury. RTP time was shorter for isolated ACL injuries and longer when associated with meniscal or cartilage injury. Given the high risk of a second ACL injury on either knee and the high reoperation rate, RTP before 5 months should be discouraged, particularly in athletes <21 years.

Anterior cruciate ligament (ACL) rupture in professional footballers represents a high burden for athletes and teams, accounting for a mean of 211 days of absence with a mean of €85,000 of economic loss for clubs in European Professional Football.^25^

Regarding the Italian First Division, the incidence of ACL injury was 0.062 × 1000 hours of play, with 14 times higher risk in matches compared with training, highlighting the increased rate of this injury in high-intensity conditions.^11^

Early ACL reconstruction (ACLR) is established as the gold standard for managing ACL ruptures in elite athletes.^6^ Evidence suggests that various grafts and surgical techniques yield favorable return to play (RTP) outcomes in elite soccer players treated with early ACLR.^2,6,7,14,30^

A recent systematic review reported an 85% return to preinjury level in elite soccer players, with a mean RTP time of 6 to 10.2 months after surgery.^15^ Similarly, a study conducted in 2021 on the Union of European Football Associations (UEFA) elite professional soccer players recorded an 80% RTP rate with significantly fewer minutes per season and recorded inferior performances for 2 seasons after injury.^9^

Recent studies on long-term clinical outcomes after ACLR have found that the mean postsurgery career length for professional footballers is between 4 and 5 years. Additionally, a decline in performance and playing time is commonly observed, with only 65% of players still competing at the top level 3 years after surgery.^16,28^

Hamstring autograft ACLR with or without a lateral extra-articular procedure (LEAP) has been shown to be a reliable surgical option; however, its application in professional soccer players has not been well studied.^3^

Lastly, a highly debated topic in literature is the rate of reoperation and graft rerupture, with recent studies reporting a 5.6% graft rerupture rate among elite soccer players, a mean time to RTP of 9.6 months after revision ACLR, and 19% soccer participation at 6 years.^15,20^

The main purpose of this study was to analyze RTP and performance of professional footballers who underwent ACLR 2 seasons after surgery to evaluate factors influencing the timing of RTP and second injury risk, and to assess the level of RTP and performance after surgery.

The hypothesis was that professional footballers undergoing ACLR with hamstring autografts, either with or without LEAP, could RTP at a high level and continue their professional careers with good long-term subjective outcomes.

## Methods

### Patients

All professional male footballers participating in the 3 main professional Italian leagues (Serie A/first division; Serie B/second division, Serie C1/third division) that underwent ACLR at our sports medicine department, Rizzoli Orthopedic Institute, Bologna, Italy, from 2002 to 2013, were retrospectively included in the study. Patients were screened from a database containing all ACLRs performed in our sports medicine department. Information about the status of professional footballers participating in the main 3 leagues was confirmed after a targeted search on publicly available databases (wikipedia.it, transfermarkt.de, and tuttocalciatori.it). Thus, the complete medical records of the eligible patients were retrieved from the department archives. Patients who underwent revision ACLR or multiligament reconstruction were excluded from the study.

### Surgical Technique and Rehabilitation

All ACLRs were performed in our sports medicine department at the Rizzoli Orthopedic Institute in Bologna, Italy, by the senior author (M.M.). Given the 10-year time interval of patient inclusion, the surgical technique of ACLR has changed according to the senior author's preference at the time of surgery, the ongoing experience, and personal considerations; the methods used were a nonanatomic double-bundle,^17^ a single-bundle over-the-top plus lateral tenodesis,^18^ and an isolated single-bundle over-the-top.^18^ However, all surgical interventions were performed using a hamstring graft while preserving the distal attachment of the hamstring tendons.

At the beginning of the procedures, a diagnostic arthroscopy was performed in all cases. In case a meniscal lesion was detected, a meniscectomy or a meniscal repair was performed depending on the extent and the localization of the lesion. All chondral lesions detected were treated with chondroplasty.

In the single bundle over the top technique, after gracilis and semitendinosus harvesting, preserving the tibial attachment, the tibial tunnel is drilled (usually 7 mm), and a straight lateral incision is performed on the lateral aspect of the knee proximal to the lateral epicondyle. By splitting the intermuscular septum, it is possible then to pierce the posterior aspect of the joint capsule with the tip of a curved Kelly clamp inserted in the anteromedial portal. In this manner, the graft is passed through the tibial tunnel, located within the joint, and then extended outside the joint through the lateral incision. The graft is tensioned manually and secured in the “over-the-top” position using two 8-mm metal staples with the knee flexed to 60° and in neutral rotation.^31^ In the single-bundle over-the-top plus lateral tenodesis technique, after fixing the graft in the over-the-top position, the remaining part of the graft is passed deep to the iliotibial band and superficial to the lateral collateral ligament. It is fixed below the Gerdy tubercle with a 6-mm metal staple as extra-articular plasty.^31^

**Figure 1.**
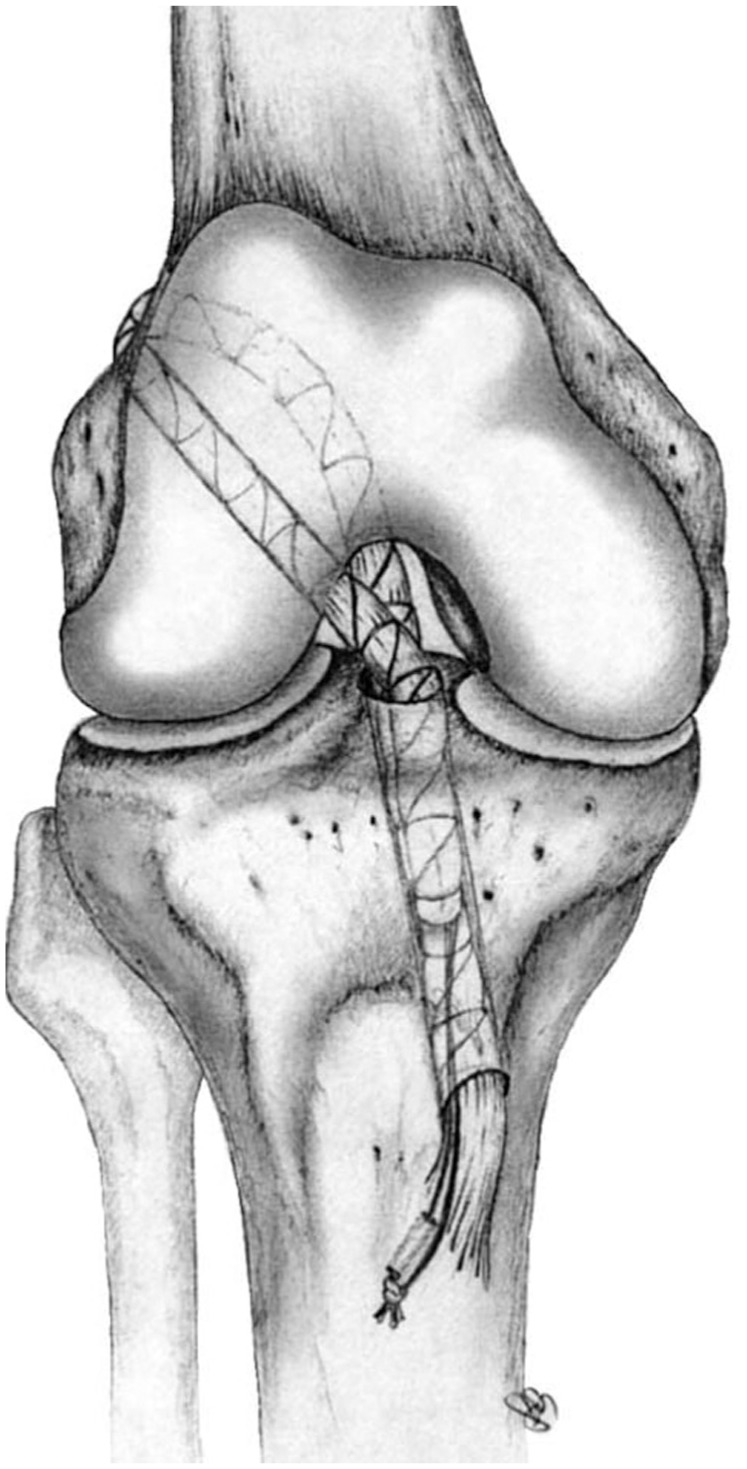
Anterior view of the knee showing the double bundle over-the-top technique with tendons passed over the top and then entering inside the femoral tunnel.

Regarding the nonanatomic double bundle, the tibial tunnel is located in the medioposterior part of the ACL tibial insertion (Figure 1). For the femoral tunnel, the knee is flexed around 130°, and the guide pin is advanced until it passes the femoral cortex. The exit point in the lateral aspect of the femur should be immediately above the end of the lateral femoral condyle. After a lateral incision, the tendons are passed over the top. Under arthroscopic view, the tendons are passed from the over-the-top position through the femoral tunnel, knee joint, and tibial tunnel to retrieve the graft from the tibial incision. The combined gracilis and semitendinosus tendons are then tensioned at 60° of flexion and in neutral position and secured with a transosseous suture knot.^17^

Due to older protocols given the cohort of patients, the aim of the age of providing a faster RTP, most frequently in the first half of the season to allow players to play before the end of the season, and the lower awareness on meniscal preserving procedures, a similar rehabilitation protocol was suggested to each athlete after surgery: no postoperative bracing since day 1, unless a meniscal repair was performed. For meniscal repair, a knee brace locked in extension is recommended for 2 to 3 weeks, depending on the severity of the lesion. Bicycle exercises and proprioceptive exercises are started 2 to 4 weeks after surgery. Running on a treadmill after 6 to 8 weeks, outside after 8 to 10 weeks. Open kinetic chain quadriceps from 90° to 40°, and isokinetic exercises for quadriceps strengthening after the sixth week. Athletes were freed to return to team training after 4 months from surgery, and to competitive sports 5 months after, and a clinical evaluation was performed by the senior authors. In some instances, based on age, contract status, ongoing competitions, and team needs, RTP was allowed earlier, typically within ≤4 months after surgery.

### Patients Evaluation

All patients signed informed consent for the surgical procedure. The study was conducted according to the principles of Helsinki, and approval was obtained from the Local Ethical Committee AVEC, Italy (protocol n° 265/2019/Oss/IOR). For all players, a targeted search was performed on the publicly available information (wikipedia.it, transkermarkt.de, tuttocalciatori.it) to extract the player characteristics and the number of matches played 2 seasons before ACL injury, the season of ACL injury, and the 2 seasons after ACL injury. Thus, a total of 5 seasons were evaluated for each player. Moreover, the level of play (first, second, third, or lower division) was determined for each of the 5 seasons. A cutoff of 20 matches in a single season was arbitrarily considered an indicator of stable playing performance,^12^ because it represents more than half of the available matches in a season, considering the regular Championship and the National/International Cups.

The same databases were screened to extract the time to RTP and the time to return to the first official match. According to Grassi et al,^10^ RTP was defined as return to team training, calculated based on the number of days absent from sport, as adopted from *transfermarkt.*^10,26^ In contrast, the return to the first official match was defined as the first appearance (in the starting line-up or entering from the bench) in an official regular match (not a friendly or second team match), according to official football charts.

Finally, hospital databases and publicly available information (Wikipedia.it, transkermarkt.de, tuttocalciatori.it, google.it) were screened to identify reinjuries, reoperations, and contralateral injuries. Patients who were reoperated on outside our institution were contacted by phone to confirm their medical history during follow-up.

### Statistical Analysis

Statistical analysis was performed with MedCalc (MedCalc Software). Continuous variables were reported as mean ± standard deviation, while categorical variables were reported as absolute number and proportion of the total sample. An independent sample *t* test was used to compare the continuous variables, and the Fisher exact test was used to compare the dichotomous categorical variables.

Kaplan-Meier survival curves were prepared using the time to RTP, time to return to official match, time to second ACL injury, and time to ipsilateral surgery as endpoints. Survival rates were calculated at 6, 12, 18, 24, 30, and 36 months.

Univariate analysis of RTP and return to official match was performed using division (first vs lower), matches played the season before injury (≤20 matches vs >20 matches), age (≥21 years vs <21 years; and ≥25 years vs <25 years), any meniscal injury, any meniscal repair, any meniscal removal, medial meniscal injury, medial meniscal repair, medial meniscal removal, lateral meniscal injury, lateral meniscal repair, lateral meniscal removal, cartilage injury, combined lateral tenodesis, and a new ipsilateral procedure within 6 months from ACLR as independent variables. Only the variables that had a *P* < .10 were used to perform a multivariate analysis. Only 1 variable related to meniscal injuries was included in the multivariate analysis, based on the *P* value of the univariate analysis and the *t* test for comparing means.

Similar univariate and multivariate analyses were performed using RTP or RTP at different time points (<5, <6, <7, <8, and <9 months), second ACL injuries (ipsilateral, contralateral, and combined) and postoperative performance (playing at least 20 matches the first season after injury, playing at least 20 matches the second season after injury, same level of play the second season after injury) as endpoints. Statistical significance was set at *P* < .05.

## Results

### Patient Characteristics

A total of 58 primary ACLRs, performed on 52 elite footballers, were included in the study. At the time of ACL injury and reconstruction, 20 footballers (34.5%) were participating in the first division (Serie A), 11 (18.9%) in the second division (Serie B), and 27 (46.6%) in the third division (Serie C1).

**Figure 2.**
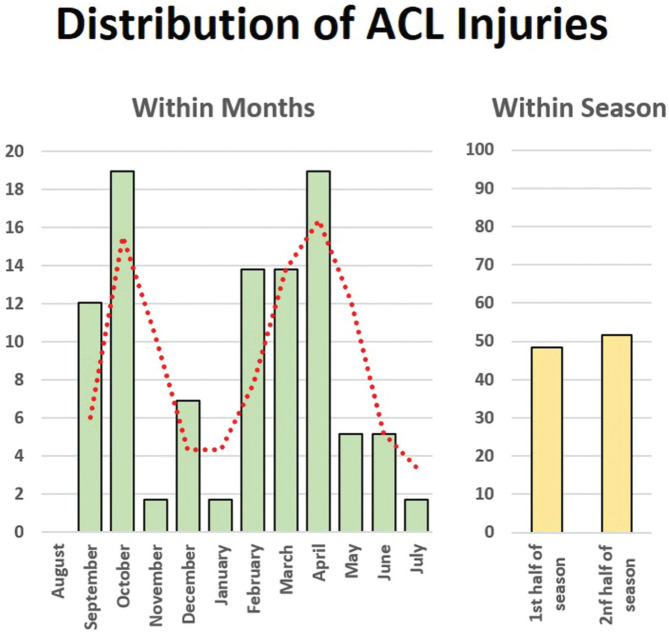
Distribution of ACL injuries during the season according to months and the first or second half of the season. ACL, anterior cruciate ligament.

The mean age at surgery was 24.9 ± 5.3 years (range, 15-39 years): 57% were <25 years old, and 26% were <21 years old at the time of surgery. Most of the included injuries occurred in October and April (Figure 2).

ACLRs were performed by the same surgeon (M.M.) on average 13 ± 12 days (range, 1-92 days) after the injury. The graft choice (autologous hamstring tendons) remained consistent throughout the period, while the surgical technique varied based on the surgeon's ongoing experience and personal considerations. Thus, a nonanatomic double-bundle was used in 35 cases (60.3%), an over-the-top single-bundle plus lateral plasty in 14 cases (24.2%), and an isolated over-the-top single-bundle technique in 9 cases (15.5%). Intact menisci were present in 25 cases (43.1%), while meniscal treatment was performed for medial meniscal injury in 16 (27.6%) cases (7 repairs, 9 partial meniscectomies) and for lateral meniscal injury in 20 (34.5%) cases (2 repairs, 18 partial meniscectomies). Medial collateral ligament retensioning was performed in 1 case (1.7%), and grade 2 or 3 Outerbridge chondral damage was noted in 5 cases (8.6%).

All the patients included in the study were followed up for 2 seasons after ACLR to detect RTP and the sport level of RTP, and the reinjury rate.

### Return to Play

**Figure 3.**
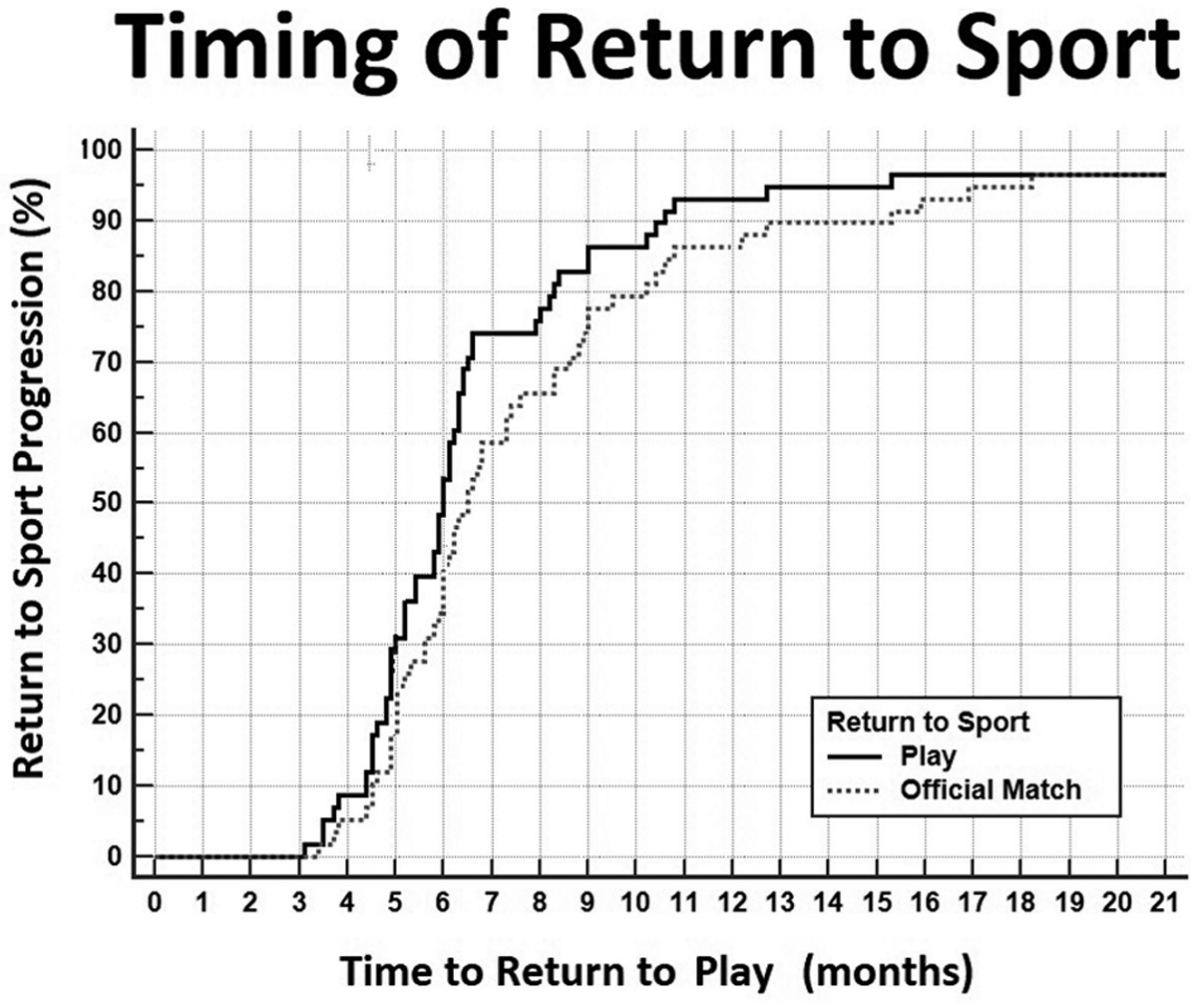
Progression of the RTP (black line) and return to official match (grey dotted line) along the postoperative months. RTP, return to play.

One 19-year-old footballer experienced an ACL rerupture before completing full rehabilitation, while another 36-year-old footballer retired after undergoing ACLR without returning to football practice. Therefore, RTP (training with the team) was achieved in 56 out of 58 cases (96.6%) at a mean of 6.4 ± 2.3 months (range, 3-15.3 months) after surgery. Subsequently, all athletes participated in an official match after returning to play with the team at a mean of 7.5 ± 3.3 months (range, 4.6-18.2 months) after surgery (Figure 3).

Nineteen cases (33.9%) returned to train with the team and played an official match during the same season as the injury, while all the other patients returned to play an official match the following season. Athletes with meniscal or cartilage injuries experienced significantly longer times to RTP and competition. Specifically, RTP was delayed to 7 ± 2.6 months for meniscal lesions (*P* = .0016) and 10.2 ± 3.7 months for cartilage lesions (*P* = .0001). Similarly, the time to return to match play was 8.1 ± 3 months for meniscal injuries (*P* = .0463) and 12.1 ± 4.9 months for cartilage injuries (*P* = .0016). Moreover, athletes who underwent a reoperation within 6 months of the index surgery had a significantly longer RTP (9.6 ± 3.2 months; *P* = .0001) and return to official match (12.2 ± 4.3 months; *P* = .0001) (Appendix Table A1).

**Figure 4.**
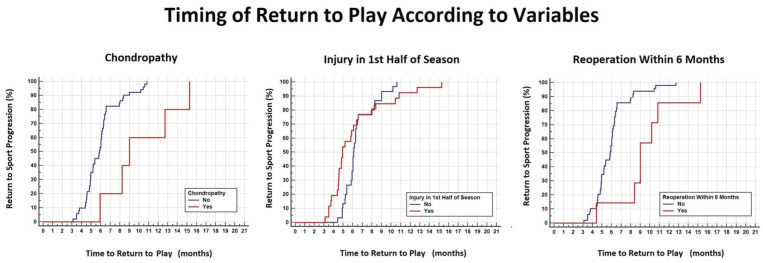
Timing of RTP according to dichotomic variables such as the presence of chondropathy (left graph), an injury during the first part of the season (center graph), and reoperation within 6 months of surgery (right graph). RTP, return to play.

According to the univariate analysis, the variables meniscal injury, cartilage injury, and reoperation within 6 months were included in the multivariate model for RTP timing (Table 1). However, only the presence of cartilage injuries was a significant predictor of a longer time to RTP (+4.4 months; SE = 1.5; *P* = .0047) (Figure 4). A similar trend was found in the multivariate model for the time to return to official match (+3.7 months; SE = 1; *P* = .0006) (Table 1).

**Table 1:** Analysis of Time to Return to Official Match*^*a*^*

	Time to RTP						Time to Return to Match					
	Univariate			Multivariate			Univariate			Multivariate		
	Coeff.	SE	*P*	Coeff.	SE	*P*	Coeff.	SE	*P*	Coeff.	SE	*P*
Meniscal injury												
Any meniscus	**1.4**	**0.6**	**.0263**	0.7	0.8	.4408	1.5	0.9	.0981	0.8	0.6	.1860
No injury												
Cartilage injury												
Present	**4.2**	**0.9**	**.0001**	**4.4**	**1.5**	**.0047**	**5.1**	**1.4**	**.0006**	**3.7**	**1.0**	**.0006**
Absent												
Reoperation within 6 months												
Yes	**3.7**	**0.8**	**.0001**	1.1	1.0	.2547	**5.4**	**1.1**	**.0001**	0.6	0.6	.3259
No												

a RTP, return to play. Bold indicates statistically significant values.

Injury in the first half of the season was a significant predictor of RTP and return to match before 5 months from injury (Figure 4, Appendix Tables A2 and A3). Indeed, footballers who had injuries in the first part of the season returned to play within the same season at a higher frequency compared with those injured in the second part (69% vs 3%; *P* = .0001). Those returning to play in the same season had a significantly shorter time to RTP (4.7 ± 1 vs 7.3 ± 2.3 months; *P* = .0018) and to return to official match (5.7 ± 1.7 vs 8.4 ± 3.4 months; *P* = .0096). A reoperation within 6 months was associated with a more extended RTP (Figure 4, Appendix Tables A2 and A3).

### Postoperative Performance

**Figure 5.**
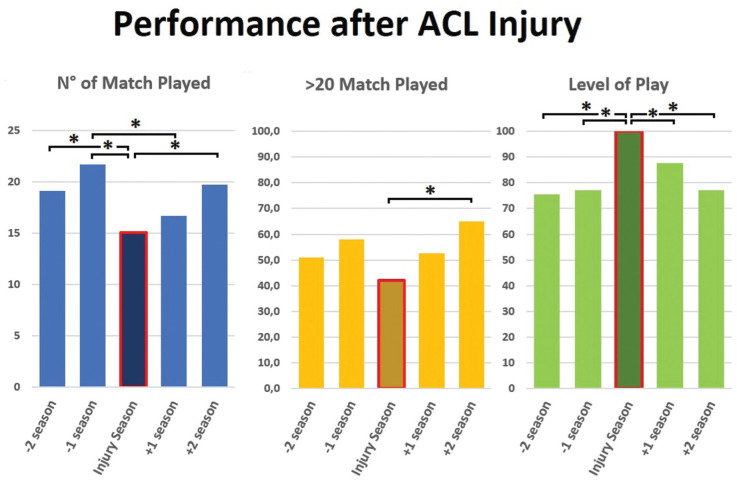
Number of matches played (blue bars), percentage of patients playing >20 matches (yellow bars), and percentage of patients playing in the same or higher division as during the season of injury (green bars) in the 2 seasons before and after injury. * refers to statistical significance.

The 36-year-old player who retired without returning to play was excluded from the performance analysis. The season before the ACL injury, 77% of footballers were competing in the same (or higher) division as the injury season and played a mean of 22 ± 10 matches, with 58% of them participating in >20 matches. The mean number of matches played during the season of injury was 15 ± 10, with only 42% of players participating in >20 matches. In the first season after the ACL injury, 12% of footballers participated in a lower division and played a mean of 17 ± 10 matches, with 53% of them participating in >20 matches. In the second season after the ACL injury, 23% of footballers participated in a lower division and played a mean of 20 ± 11 matches, with 65% of them participating in >20 matches (Figure 5).

The number of matches played during the injury season was significantly lower with respect to the second (*P* = .0479) and the first (*P* = .0007) seasons before injury. An improvement in match participation, with a return to values similar to those before the injury, was observed only in the second season after the ACL injury (*P* = .0220). Moreover, the percentage of players with >20 matches was significantly higher in the second season after ACL injury with respect to the injury season (*P* = .0231) (Figure 5).

RTP or to match before 8 months from injury were the variables with higher odds ratios to participate in at least 20 matches the first season after ACL injury (Appendix Table A4). No predictors were identified for participating in at least 20 matches in the second season after an ACL injury or competing at the same preinjury level.

### Second ACL Injuries and Reoperations

**Figure 6.**
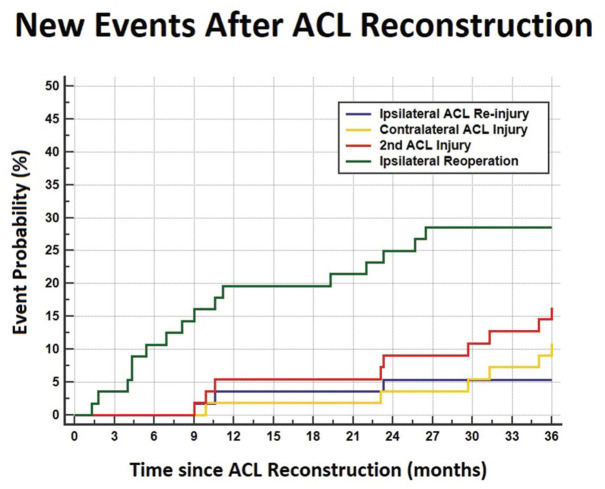
Events that occurred during the considered follow-up included ipsilateral ACL reinjury (blue line), contralateral ACL injury (yellow line), second ACL injury (red line), or overall ipsilateral reoperation (green line). ACL, anterior cruciate ligament.

In addition to the 19-year-old footballer who experienced graft rerupture 9 months after ACLR before RTP, 2 other players, aged 17 and 25 years, experienced ACL failure within the first 3 seasons after ACLR, at 23 months and 11 months, respectively (Figure 6).

Thus, the overall failure rate within the first 3 seasons after ACLR was 5.3%, with no significant differences among the 3 techniques. A contralateral ACL injury occurred in 6 patients, on average 27 ± 10 months after surgery and 22 ± 9 months after returning to play, yielding a contralateral injury rate of 10.5%. Thus, a total of 9 athletes incurred an ipsilateral rerupture or contralateral injury, accounting for an overall second ACL injury rate of 15.8% (Table 2).

**Table 2:** Rate of Ipsilateral ACL Revision, Contralateral ACL Injury, Overall Second ACL Injury, and Overall Ipsilateral ACL Reoperations at Different Time Points*^*a*^*

Outcome	6 Months	12 Months	18 Months	24 Months	30 Months	36 Months
Ipsilateral ACL revision	0	3.5	3.5	5.3	5.3	5.3
Contralateral ACL injury	0	1.8	1.8	3.5	5.3	10.5
Second ACL injury	0	5.3	5.3	8.8	10.5	15.8
Overall ipsilateral reoperation	10.5	19.6	19.6	25	28.1	28.1

a Data are presented as %. ACL, anterior cruciate ligament.

Excluding the 3 ipsilateral ACL revisions, the 13 other footballers underwent an ipsilateral reoperation within the 3 seasons after ACLR, after a mean of 11 ± 9 months. The procedures included 5 medial meniscectomies (2 cases had a previous repair), 2 autologous chondrocyte implantations, 2 arthroscopic arthrolysis, 1 lateral meniscus transplantation, 1 microfracture, 1 loose body removal, and 1 hardware removal. Therefore, the overall ipsilateral reoperation rate was 22.8%, which increased to 28.1% when considering ACL revisions as well (Table 2). All but 3 reoperations were conducted at our institution; the other 3 patients were contacted telephonically to assess reoperations.

### Analysis of RTP and Second ACL Injuries

**Figure 7.**
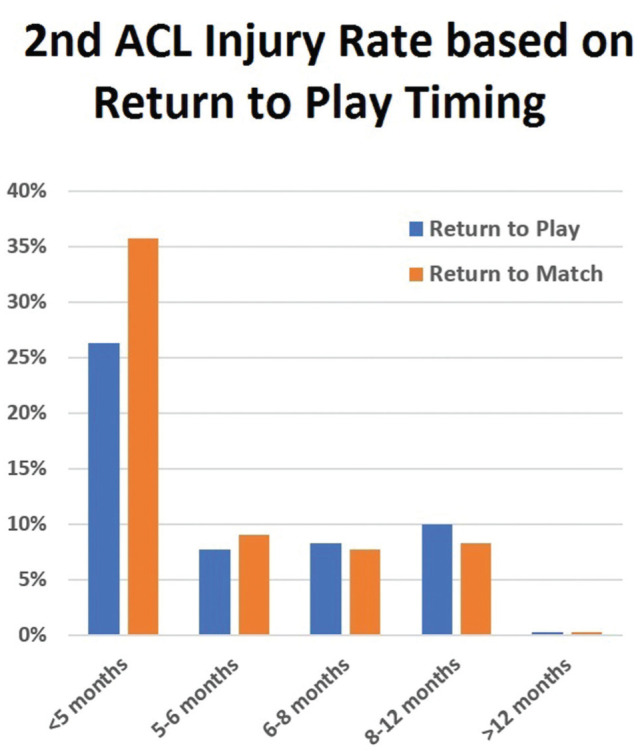
Rate of the second ACL reinjury stratified according to the timing of RTP (blue bar) or return to official match (orange bar). ACL, anterior cruciate ligament; RTP, return to play.

According to the univariate analysis, returning to an official match within 5 months from injury was the only significant predictor of a second ACL injury (Table 3). A significant difference was observed in the second ACL injury rate among athletes who returned to match play within 5 months (36%) and those who returned after 5 months (7%) from injury (Appendix Table A5). On the other hand, the second ACL injury rate was similar in athletes returning to play or match between 5 to 6 months, 6 to 8 months, and 8 to 12 months (Figure 7).

**Table 3:** Univariate and Multivariate Analysis for Predictors of Second ACL Injury*^*a*^*

		Univariate			Multivariate Model 1			Multivariate Model 2			Multivariate Model 3		
					(RTP <5 Months)			(Return to Match <5 Months)			(Return to Match <6 Months)		
		HR	95% CI	*P*	HR	95%CI	*P*	HR	95%CI	*P*	HR	95%CI	*P*
Age <21 years		3.4	(0.9-14.9)	.0614	**5.6**	**(1.4-22.7)**	**.0177**	4.0	(1- 16.3)	.0528	**4.2**	**(1-16.8)**	**.0442**
Any meniscal injury		0.2	(0-1.1)	.0617	0.3	(0.1-1.5)	.1344	0.4	(0.1- 2.4)	.3323	**0.2**	**(0.1-1)**	**.0451**
RTP <5 months		4.0	(1-16.8)	.0563	4.2	(0.9-19.1)	.0645	NI			NI		
Return to match <5 months		**11.9**	**(2.4-58.7)**	**.0025**	NI			**9.4**	**(1.7- 5.2)**	**.0107**	NI		
Return to match <6 months		4.5	(0.9-22.3)	.0647	NI			NI			4.7	(1-23.5)	0.0557

a Different multivariate models have been used with different RTP and return to match timing. ACL, anterior cruciate ligament. HR, hazard ratio; NI, not included; RTP, return to play. Bold indicates statistically significant values.

According to the multivariate analysis, which included variables such as RTP before 5 months, return to match before 5 months, or return to match before 6 months, the statistically significant predictors were returning to match before 5 months, as in univariate analysis, age at surgery, <21 years, and the absence of meniscal injuries (Figure 7, Table 3). When combining the 2 main predictors of the second ACL injury, which have higher hazard ratios (return to match before 5 months and age <21 years), the overall rate was 60% (Table 4).

**Table 4:** Rate of Second Injuries Stratified According to Return to Official Match and Age at Surgery*^*a*^*

Return to match <5 months and age <21 years	3/5 (60)
Return to match <5 months and age ≥21 years	3/10 (30)
Return to match ≥5 months and age <21 years	1/9 (11)
Return to match ≥5 months and age ≥21 years	1/32 (3)
*P*	**.0031**

a Data are presented as n/n (%). The bold *P* value indicates significance.

## Discussion

The most important finding of the present study was that RTP after ACLR in elite male footballers was close to 100% using hamstring autografts, with or without LEAP. Moreover, the presence of meniscal injuries and cartilage lesions was correlated with delayed RTP. Regarding sports performance in the first 2 years after returning to play, a decreased level was noted in nearly 20% of athletes. However, a recovery in the number of matches played compared with the preinjury status was observed in the season after surgery. Furthermore, a second ACL injury was reported in 15.8% of patients, with rates as high as 30% in athletes <21 years and in those returning to match play within 5 months. Finally, the overall ipsilateral reoperation rate at 36 months was 22.8%, which increased to 28.1% when considering ACL revisions as well.

The demographic characteristics of the elite male footballers included in the present study are similar to those in other studies on comparable populations. The mean age at surgery of 24.9 years is consistent with the 23.1 years reported in a series of 27 European footballers,^8^ the 25.2 years in a series of 82 top-level English players,^2^ and the 25 years observed in 118 players from various UEFA clubs.^5^

Moreover, the number of concomitant meniscal lesions exceeding 50% of cases was similar to that in other studies involving professional soccer players.^8^ These findings confirm the recurrent demographics and injury patterns among male footballers participating in top-level European leagues, supporting a high degree of external validity of the evidence related to this specific patient subgroup.

The data regarding the RTP after ACLR in elite male footballers reported in the present study confirms a trend already reported in the literature for this specific group of patients. In fact, Della Villa et al^5^ reported a rate of 98% RTP in players from various UEFA leagues, while Farinelli et al^8^ reported a rate of 92.6% in European elite footballers; these data are consistent with the 97% reported in the present study, independently of the surgical characteristics and graft choice. It should be emphasized that RTP for professional football in male athletes is highly effective, with a success rate of nearly 100%. This should not be taken for granted, as inferior rates have been reported in other sports with different technical and athletic requirements, such as basketball (84%)^4^ and American football (55.8%).^22^

Regarding the timing of RTP, interesting conclusions could be drawn from the multivariate analysis. The shorter time for RTP was reported in patients with no meniscal or chondral injuries. This seems a reasonable finding because the lack of a concomitant procedure could have allowed an accelerated and faster rehabilitation. Despite the more optimistic scenario in the absence of concurrent lesions, extreme caution should be exercised to avoid excessively speeding up RTP, as Della Villa et al^5^ reported a higher rate of second ACL injuries in professional footballers with isolated ACL injuries. Conversely, a longer time to RTP was found in athletes with concomitant cartilage injuries. This is an important factor to consider when establishing a proper prognosis with the athlete, the coach, and the team at the beginning of injury management.

Another interesting finding of the present study is the timing of RTP of 6.4 months for full practice and 7.5 months for the first official match. This timing seems relatively shorter compared with the mean of 8 to 10 months^1,8^ reported by other authors managing professional footballers. In this regard, it should be acknowledged that the approach of the senior surgeon at the time of patient treatment (2002-2013) was to speed up the RTP in those athletes who sustained ACL injury in the first part of the season, usually within the Christmas break typical of European leagues. Considering the 10-month football season, which spans from August to May, a significantly shorter RTP was reported in those injured in the first part of the season (5.7 months vs 8.4 months). This aspect should be considered during the initial management of ACL injury when establishing a reliable prognosis for RTP by balancing the risk of a second injury with the possibility of participating in relevant competitions (World Cup, European Championship, Champions League Final, etc) during the spring-summer, the years of contract, and possibly other future prospectives.

While our data suggest slightly shorter return-to-train and return-to-match times after meniscectomy compared with meniscal repair (6.9 vs 7 months and 7.9 vs 9.2 months, respectively), these differences did not reach clinical significance. Moreover, no significant difference was observed in the overall RTP rates between the 2 procedures. This may be partly explained by the rehabilitation protocols, which, after the initial few weeks, were largely similar for both meniscectomy and meniscal repair, aiming for an expedited RTP, particularly in the early part of the season. Additionally, the possibility of a type 2 error due to the relatively small sample size cannot be excluded, and may have limited our ability to detect a statistically significant difference.

The analysis of performance after ACLR and RTP was possible only for the first 2 seasons after surgery and was limited to the level of play and the number of matches played. A cutoff of 20 games played was selected for our cohort because it represents approximately half of the average number of games (40) played in a full season, including matches from the National Championship as well as National and International Cups. Furthermore, unlike in sports such as American baseball, professional soccer players typically return to the same division after ACL injury. Therefore, returning to play in a lower division is considered a negative indicator of postinjury performance. No specific performance indicators, such as minutes played, goals, assists, or others, were possible based on the available data. While nearly 100% of footballers returned to play, 12% to 23% of them did so in a lower category, thus highlighting the possible effect of this injury on the athlete's career. In fact, Borque et al^2^ demonstrated a 1.6-year career shortening in professional footballers who sustained an ACL injury, while Niederer et al^23^ reported a lower level of play and worse performances compared with a cohort of uninjured players. Further studies should be conducted in this regard, utilizing improved performance indicators and incorporating more football-specific factors, such as market value, contract duration, and career prospects.

Regarding the number of games played in the season after ACLR, 53% of the soccer players played >20 games. We considered this a successful RTP, as the result aligns with the findings in the literature. A recent study on 195 elite professional male soccer players has shown a significant reduction in minutes played per season compared with the preoperative season across all age groups.^21^ Moreover, preoperatively, 57% of footballers were playing >20 games per season, thus indicating only a slight reduction in performance and a satisfactory level of RTP in the first season after ACLR.

The rate of reruptures requiring revision was low in this study, despite the use of a hamstring graft, which some surgeons do not recommend for professional athletes.^3^ The rate of 5.3% is similar to the 6.7% by Balendra et al,^1^ using bone-patellar tendon-bone (BPTB), or to the 7.4% using quadriceps tendon or BPTB by Farinelli et al,^8^ in similar populations of Elite professional European footballers. This could be because, in most cases, either a double-bundle technique or a lateral tenodesis was performed. Moreover, preserving the hamstring tibial insertion could have improved ligament healing, as demonstrated by in vitro and clinical studies.^31^ The efficacy of the present surgical approach was also confirmed by the higher rate of contralateral ACL injury (10.5%) with respect to the ipsilateral rerupture (5.3%).

The overall 15.8% rate of second ACL injury (either ipsilateral or contralateral) raises important considerations, particularly regarding RTP timing.

Interestingly, the rate of contralateral ACL injury was higher than that of ipsilateral reinjury. No evidence demonstrates the reason why patients who undergo ACLR are at a higher risk of injuring the contralateral ACL. However, a previous study^29^ hypothesized that the increased risk may be determined by a tendency to protect the operated side, which could result from inadequate rehabilitation of the contralateral knee that was deconditioned during recovery from the ipsilateral knee surgery. More long-term prospective longitudinal studies will need to be performed to identify predictors for these contralateral injuries.

Despite the general concept of high reinjury risk in early RTP,^13^ a recent systematic review demonstrated that for professional athletes, there is no significant difference in time to RTP between those who suffered a second ACL injury and those who did not.^24^ In the present study, the timing of RTP was considered a risk factor for reinjury only in the case of return to an official match before 5 months, while similar reinjury rates were reported in those returning to play or match after 5 months. Moreover, the stratified analysis based on players’ age highlighted a higher risk of a second injury in athletes <21 years, with a rate that reached 60% in those returning to match before 5 months.

Thus, RTP should be delayed in younger patients, and RTP within the same season in these patients should be strongly discouraged. However, based on the limited retrospective data from this cohort and the relevant confounding factors, an ideal timing for RTP aimed at reducing the reinjury rate could not be provided.

The overall reoperation rate of 22.8% excluding ipsilateral ACL revisions, is relevant and offers important points for discussion. This result may be influenced by the early RTP observed in this patient cohort, driven by high functional demands and the need to resume sports activity as soon as possible after injury. Such a premature return may not allow for a complete restoration of biomechanics, particularly in the presence of associated lesions, in a context where patients undergo intensive rehabilitation primarily aimed at returning to play. The literature supports this hypothesis: a study by Török et al^27^ found a statistically significant correlation between the rate of reoperations and the type of rehabilitation protocol (conventional vs accelerated) after ACLR. On the other hand, a recently published study on a similar competitive population yielded a 24% reoperation rate,^19^ highlighting also the fact that the high rate of subsequent surgery could be caused by the high functional requests of those patients and high level of performance.

### Limitations

This study has several limitations. First, it is a retrospective study. Moreover, the surgical technique has evolved over time, resulting in a heterogeneous cohort of patients, which makes it challenging to analyze. However, this study demonstrates the validity of all 3 techniques with a low failure rate and a high percentage of RTP at a high level.

Furthermore, this study does not evaluate the outcomes of different grafts for ACLR in professional athletes. However, the main aim of this study was to assess RTP after ACLR with various over-the-top techniques using hamstring autografts, thus representing a novelty in the existing literature.

Another limitation of this study is the follow-up time, which takes into account only the 2 seasons after the injury, avoiding the evaluation of long-term outcomes such as career length and incidence of osteoarthritis. However, this study was focused on RTP, factors influencing RTP, and the level of performance of players after ACLR.

Furthermore, the sample size is relatively limited compared with other studies evaluating similar outcomes. However, this cohort of patients, representing professional soccer players from elite divisions in Italy, represents one of the largest cohorts of its kind in the literature regarding soccer players in Italy.

Another significant limitation is the presence of the same rehabilitation protocol throughout all the patients, with only slight differences in the return to run, which, on the one hand, makes a homogeneous evaluation of the patients easier but on the other hand could represent some bias in the reoperation rate or in the level of performance due to inadequate rehabilitation in patients with relevant associated lesions.

Lastly, another limitation is the absence of strict RTP criteria, such as isokinetic tests and readiness tests for performance. However, the evaluation of RTP clearance has been made by the senior author, in collaboration with the medical staff team, on a case-by-case basis, depending on the patient's level of preparation and associated lesions.

## Conclusion

Almost all patients RTP after ACLR using a hamstring graft with over-the-top techniques, typically regaining their preinjury performance by the second season after the injury. RTP time was shorter for isolated ACL injuries and longer when associated with meniscal or cartilage injury. Given the high percentages of a second ACL injury on either knee and the high reoperation rate, RTP before 5 months should be discouraged, particularly in athletes aged <21 years. However, more prospective studies are needed to determine the optimal timing for returning to play.

## Appendix

**Appendix Table A1:** Analysis of Time to RTP and Match*^*a*^*

	Footballers		Time to RTP		Time to Return to Match	
	N	%	Months	*P*	Months	*P*
Division				
First division, series A	19	(33.9)	6.1 ± 2.3	NS	7.6 ± 3.2	NS
Lower divisions, B and C1	37	(66.1)	6.5 ± 2.4		7.4 ± 3.4	
No. of matches before injury				
>20 matches	32	(57.1)	6 ± 2	NS	7.1 ± 3.0	NS
≤20 matches	24	(42.9)	6.8 ± 2.7		7.9 ± 3.4	
Age <21 years						
<21	14	(25)	6.8 ± 2.6	NS	7.9 ± 3.4	NS
≥21	42	(75)	6.3 ± 2.3		7.4 ± 3.2	
Age <25 years						
<25	31	(55.4)	6.4 ± 2.2	NS	7.7 ± 3.4	NS
≥25	25	(44.6)	6.3 ± 2.6		7.2 ± 3.3	
Injury timing in season						
First half of the season	26	(46.4)	6.2 ± 3	NS	7.3 ± 3.6	NS
Second half of the season	30	(53.6)	6.5 ± 1.6		7.6 ± 3.1	
Meniscal injury						
Any meniscus	32	(57.1)	**7 ± 2.6**	**.0016**	**8.1 ± 3**	**.0463**
No injury	24	(42.9)	**5.6 ± 1.6**		**6.6 ± 2.3**	
Meniscal removal						
Any meniscus	23	(41.1)	6.9 ± 2.7	NS	7.8 ± 3.9	NS
No removal	33	(58.9)	6 ± 2.1		7.2 ± 2.9	
Meniscal repair						
Any repair	11	(19.6)	7 ± 2.6	NS	9.2 ± 4.2	NS
No repair	45	(80.4)	6.2 ± 2.3		7.1 ± 2.9	
Medial meniscal injury						
Yes	16	(28.6)	7.3 ± 3.1	NS	8.7 ± 4.1	NS
No injury	40	(71.4)	6 ± 1.9		7.0 ± 4.2	
Medial meniscal removal						
Yes	7	(12.5)	7.9 ± 3.8	NS	8.1 ± 3.7	NS
No removal	49	(87.5)	6.2 ± 2		7.4 ± 3.3	
Medial meniscal repair						
Yes	9	(16.1)	6.7 ± 2.5	NS	9.2 ± 4.7	NS
No repair	47	(83.9)	6.3 ± 2.3		7.2 ± 2.9	
Lateral meniscal injury						
Yes	19	(33.9)	6.7 ± 2.1	NS	7.9 ± 3.4	NS
No injury	37	(66.1)	6.2 ± 2.5		7.2 ± 3.1	
Lateral meniscal removal						
Yes	17	(30.4)	6.6 ± 2	NS	7.8 ± 3.9	NS
No removal	39	(69.6)	6.3 ± 2.5		7.4 ± 3.1	
Lateral meniscal repair						
Yes	2	(3.6)	8.3 ± 3.5	NS	9.0 ± 2.5	NS
No repair	54	(96.4)	6.3 ± 2.3		7.4 ± 3.3	
Cartilage injury						
Present	4	(7.1)	**10.2 ± 3.7**	**.0001**	**12.1 ± 4.9**	**.0016**
Absent	52	(92.9)	**6 ± 1.8**		**7 ± 2.8**	
Lateral tenodesis						
Present	14	(25)	6.8 ± 3.1	NS	7.8 ± 4.1	NS
Not performed	42	(75)	6.2 ± 2.1		7.4 ± 3.0	
Reoperation within 6 months						
Yes	7	(12.5)	**9.6 ± 3.2**	**.0001**	**12.2 ± 4.3**	**.0001**
No	49	(87.5)	**5.9 ± 1.8**		**6.8 ± 2.7**	

a Data are presented as n (%) and mean ± SD, unless otherwise indicated. NS, not significant. RTP, return to play. Bold indicates statistically significant values.

**Appendix Table A2:** Predictors of RTP at Different Time Points*^*a*^*

	Rates		Univariate			Multivariate		
RTP <5 Months	%	*P*	HR	95% CI	*P*	HR	95% CI	*P*
Injury timing in the season								
First half of the season	54	**.0033**	4.5	(1.6-12.6)	**.0039**	4.8	(1.7-13.1)	**.0030**
Second half of the season	17							
Medial meniscal injury								
Yes	19	NS	0.5	(0.2-1.1)	.0850	0.4	(0.1-1.3)	.1306
No injury	40							
RTP <6 Months		*P*	HR	95% CI	*P*	HR	95% CI	*P*
Reoperation within 6 months								
Yes	14	**.0347**	0.2	(0.1-1.2)	.0782	NP		
No	63							
RTP <7 Months	%	*P*	HR	95% CI	*P*	HR	95% CI	*P*
Cartilage injury								
Present	25	**.0083**	0.1	(0.1-1)	**.0486**	0.2	(0.1-1.4)	.0987
Absent	81							
Reoperation within 6 months								
Yes	14	**.0086**	0.1	(0.1-0.7)	**.0181**	0.1	(0.1-0.8)	**.0380**
No	86							
RTP <8 Months	%	*P*	HR	95% CI	*P*	HR	95% CI	*P*
Cartilage injury								
Present	25	**.0059**	0.1	(0.1-0.9)	**.0426**	0.2	(0-1.2)	.0861
Absent	84							
Reoperation within 6 months								
Yes	14	**.0086**	0.1	(0-0.7)	**.0149**	0.1	(0-0.8)	**.0253**
No	86							
RTP <9 Months	%	*P*	HR	95% CI	*P*	HR	95% CI	*P*
Cartilage injury								
Present	75	**.0835**	0.3	(0.1-0.9)	**.0400**	0.4	(0.1-1.1)	.0846
Absent	90							
Reoperation within 6 months								
Yes	57	**.0212**	0.2	(0.1-0.6)	**.0053**	0.2	(0.1-0.8)	**.0103**
No	94							

a HR, hazard ratio; NP, not performed; RTP, return to play. Bold indicates statistically significant values.

**Appendix Table A3:** Predictors of Return to Official Match at Different Time Points*^*a*^*

	Rates		Univariate			Multivariate		
Return to match <5 Months	%	*P*	HR	95% CI	*P*	HR	95% CI	*P*
Injury timing in the season								
First half of the season	42	**.0183**	4.0	(1.3-12.5)	**.0177**	3.5	(1.1-11)	**.0349**
Second half of the season	13							
Meniscal injury								
Any meniscus	16	**.0374**	0.3	(0.1-1)	**.0405**	0.4	(0.1-1.2)	.1070
No injury	42							
Medial meniscal injury								
Yes	6	**.0431**	0.2	(0.1.1)	.0695	NI		
No injury	35							
Return to Match <6 Months		*P*	HR	95% CI	*P*	HR	95% CI	*P*
Meniscal repair								
Any repair	18	.0883	0.3	(0.1-1.2)	.0833	NP		
No repair	51							
Return to Match <7 Months	%	*P*	HR	95% CI	*P*	HR	95% CI	*P*
Reoperation within 6 months								
Yes	14	**.0116**	**0.1**	**(0-1)**	**.0490**	NP		
No	67							
Return to Match <8 Months	%	*P*	HR	95% CI	*P*	HR	95% CI	*P*
Cartilage injury								
Present	25	**.0326**	0.2	(0-1.3)	.0924	0.3	(0-1.9)	.1802
Absent	71							
Reoperation within 6 months								
Yes	14	**.0031**	0.1	(0-0.8)	**.0304**	**0.1**	**(0-1)**	**.0477**
No	76							
Return to Match <9 Months	%	*P*	HR	95% CI	*P*	HR	95% CI	*P*
Cartilage injury								
Present	50	**.0467**	0.1	(0-0.8)	**.0304**	0.3	(0-1.9)	.1802
Absent	83							
Reoperation within 6 months								
Yes	29	**.0020**	0.2	(0-1.4)	.0924	**0.1**	**(0-1)**	**.0477**
No	88							

a HR, hazard ratio; NI, not included; NP, not performed. Bold indicates statistically significant values.

**Appendix Table A4:** Performances After ACL Injury*^*a*^*

At least 20 Matches 1st Season after ACL Injury				
		OR	95% CI	*P*
RTP	Months	0.6	(0.4-0.8)	.0006
	<5	NS		
	<6	NS		
	<7	5.6	(1.3-23.5)	.0180
	<8	8.7	(1.7-45.9)	.0094
	<9	NS		
Return to Official Match	Months	0.6	(0.4-0.8)	.0014
	<5	9	(1.9-46)	.0071
	<6	4.1	(1.2-40.7)	.0152
	<7	6.4	(1.9-21.1)	.0023
	<8	12.3	(3-51)	.0006
	<9	NS		
At least 20 Matches Second Season After ACL Injury				
		OR	95% CI	*P*
All variables		NS		
Same level of play, Second Season After ACL Injury				
		OR	95% CI	*P*
All variables		NS		

a ACL, anterior cruciate ligament; NS, not significant; OR, odds ratio; RTP, return to play.

**Appendix Table A5:** Ipsilateral, Contralateral, and Second ACL Injuries According to RTP and Return to Match Timing*^*a*^*

	Footballers		Ipsilateral ACL		Contralateral ACL		Second ACL Injury	
RTP, Months	N	%	Rate	*P*	Rate	*P*	Rate	*P*
5								
<5	19	34	5	NS	21	NS	26	NS
≥5	37	66	3		5		8	
6								
<6	32	57	6	NS	13	NS	19	NS
≥6	24	43	0		8		8	
7								
<7	43	77	5	NS	12	NS	16	NS
≥7	13	23	0		8		8	
8								
<8	44	79	5	NS	11	NS	16	NS
≥8	12	21	0		8		8	
9								
<9	50	89	4	NS	10	NS	14	NS
≥9	6	11	0		17		17	
Return to Match, Months	N	%	Rate	*P*	Rate	*P*	Rate	*P*
5								
<5	14	25	7	NS	29	NS	36	**.0181**
≥5	42	75	2		5		7	
6								
<6	24	43	4	NS	17	NS	21	NS
≥6	32	57	3		6		9	
7								
<7	34	61	6	NS	15	NS	21	NS
≥7	22	39	0		5		5	
8								
<8	38	68	5	NS	13	NS	18	NS
≥8	18	32	0		6		6	
9								
<9	45	80	4	NS	11	NS	16	NS
≥9	11	20	0		9		9	

a ACL, anterior cruciate ligament; NS, not significant; RTP, return to play. Bold indicates statistical significance.
